# Supplementary material for: Characterising open chromatin in chick embryos identifies cis-regulatory elements important for paraxial mesoderm formation and axis extension
Source: Nat Commun. 2021 Feb 19;12:1157. doi: 10.1038/s41467-021-21426-7 (PMC7895974; doi:10.1038/s41467-021-21426-7)
Supplement: Supplementary file 1 — Supplementary Information [file 41467_2021_21426_MOESM1_ESM.pdf]

Supplementary Figures and legends

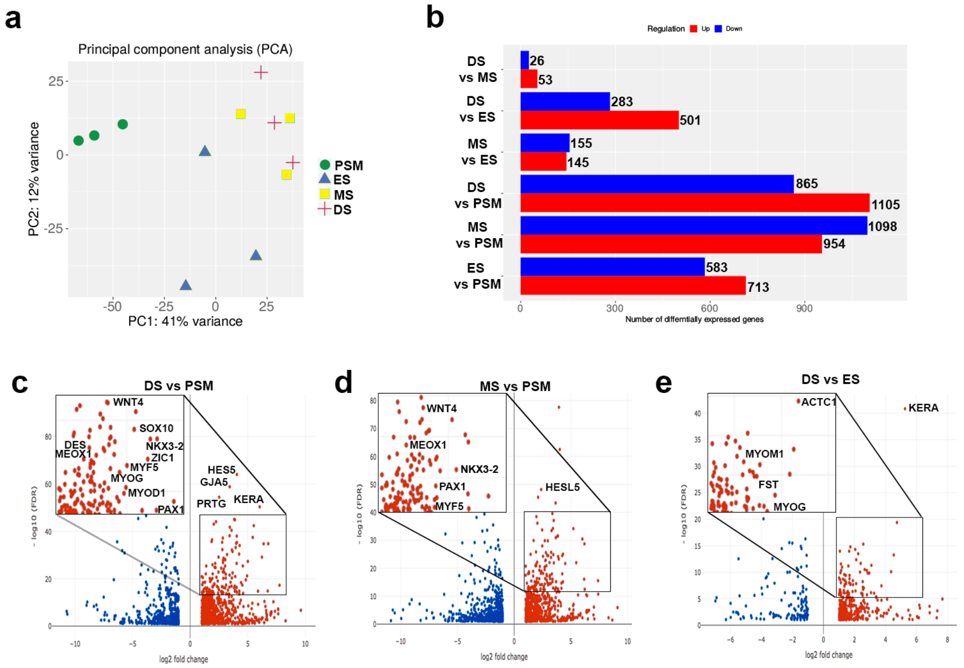

f

Cluster	adj.Pval	nGenes	Pathways
B	1.68E-12	56	Anatomical structure morphogenesis
B	1.20E-09	40	Ion transport
B	1.31E-08	66	Cellular developmental process
C	1.62E-17	74	Anatomical structure morphogenesis
C	3.13E-14	60	Cell development
C	1.26E-12	41	Cellular component morphogenesis
D	1.78E-16	59	Cell differentiation
D	5.65E-16	47	Anatomical structure morphogenesis
D	1.89E-15	41	Regulation of multicellular organismal development
F	6.79E-08	8	Cholesterol homeostasis
F	3.45E-07	25	Small molecule metabolic process
F	3.97E-06	12	Organic hydroxy compound metabolic process
G	3.26E-08	77	Animal organ development
G	3.08E-07	61	Anatomical structure morphogenesis
G	8.26E-07	81	Cell differentiation
H	2.08E-15	10	Embryonic limb morphogenesis
H	2.28E-15	13	Pattern specification process
H	4.25E-15	12	Regionalization
I	1.32E-10	67	Regulation of cell communication
I	2.56E-10	62	Regulation of signal transduction
I	2.75E-10	54	Anatomical structure morphogenesis
J	0.003382062	14	Cell morphogenesis involved in differentiation
J	0.005563767	5	Positive regulation of blood circulation
J	0.007734162	28	Anatomical structure morphogenesis

Supplementary Fig. 1: Transcriptional profiling of developing somites. a PCA plot of PSM, ES, MS and DS replicates. **b** Number of differentially expressed (upregulated, red; downregulated, blue) genes comparing PSM, ES, MS and DS. **c-e** Volcano plots showing enriched genes (Log Fold Change >1) comparing DS vs PSM, (**d**) MS vs PSM and (**e**) ES vs PSM. **f** GO analysis on k-means cluster showing associated terms, adjusted P-values and number of genes in each associated term.

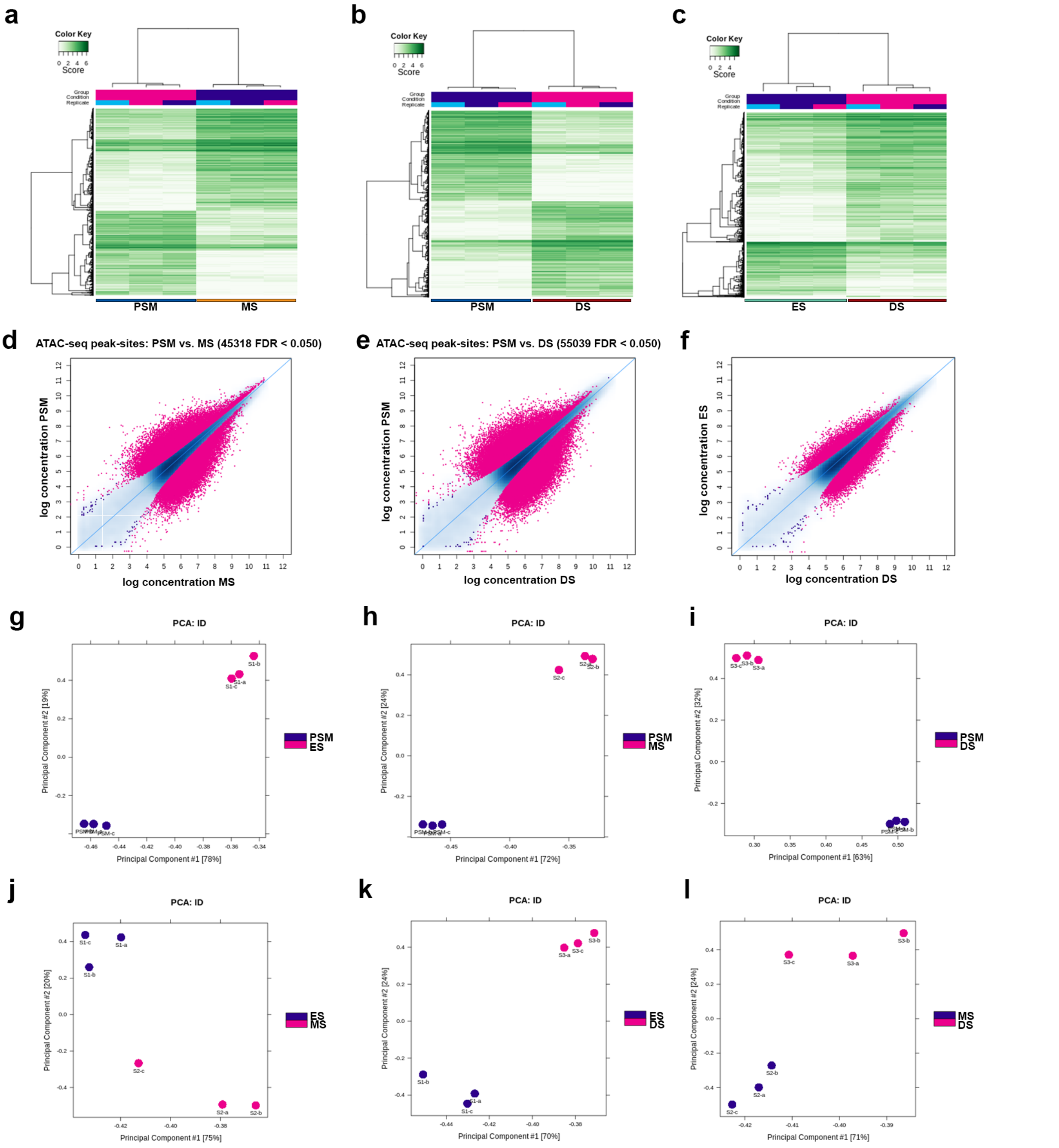

Supplementary Fig. 2: Genome-wide profile of chromatin accessibility dynamics during somite development. a Correlation heat maps of accessible chromatin regions (ATAC-seq peak-sites) comparing PSM and MS, (**b**) PSM and DS and (**c**) ES and DS. **d** MA plots of significantly differential peak-sites in PSM with MS, (**e**) ES with DS and (**f**) ES with DS. **g-l** PCA plots using only the differentially accessible peak-sites using an FDR threshold of 0.05.

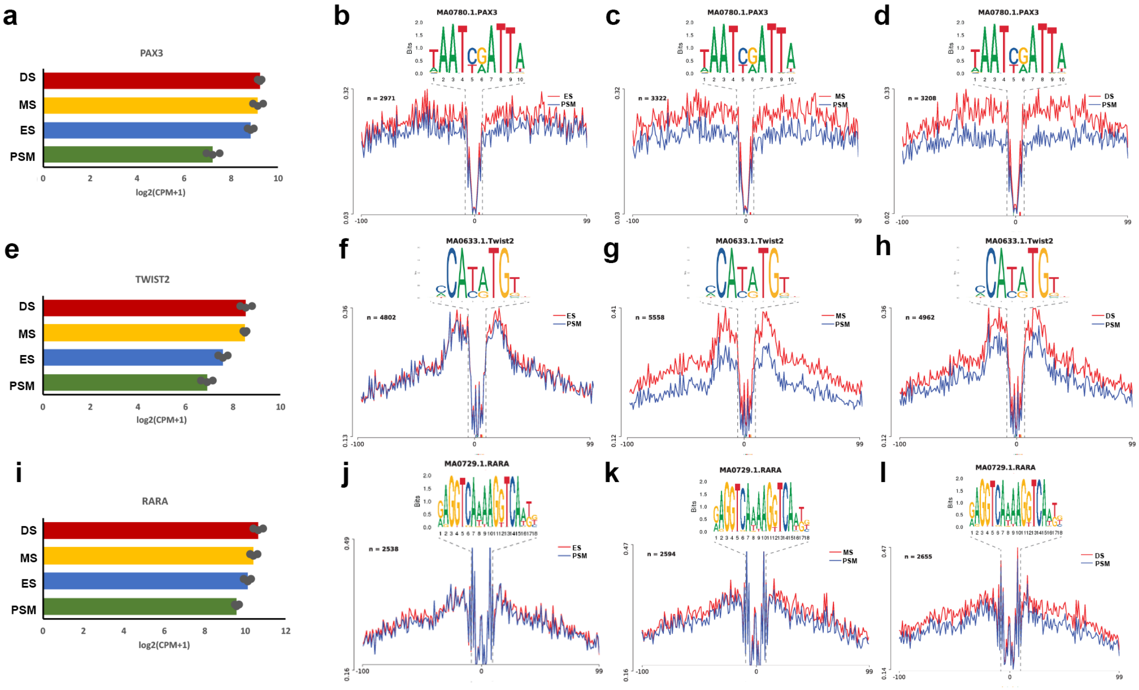

Supplementary Fig. 3: Differential footprints identified for key paraxial mesoderm transcription factors. a Gene expression for *Pax3* from mRNA-seq (error bars = SEM, n = 3). **b** Tn5 insertion frequency across all accessible regions containing at least one Pax3 motif, at nucleotide resolution in PSM, ES, MS and DS reveals presence of a footprint centered on the Pax3 motif. Differential footprinting for Pax3 motif comparing PSM (blue) and ES (red), (**c**) PSM (blue) and MS (red) and (**d**) PSM (blue) and DS (red). **e** Gene expression for *Twist2* from mRNA-seq (error bars = SEM, n = 3). **f** Differential footprinting for Twist2 motif comparing PSM (blue) and ES (red), (**g**) PSM (blue) and MS (red) and (**h**) PSM (blue) and DS (red). **i** Gene expression for *RARA* from mRNA-seq (error bars = SEM, n = 3). **j** Differential footprinting for RARA motif comparing PSM (blue) and ES (red), (**k**) PSM (blue) and MS (red) and (**l**) PSM (blue) and DS (red). The total number of footprints detected is shown in each panel, n.

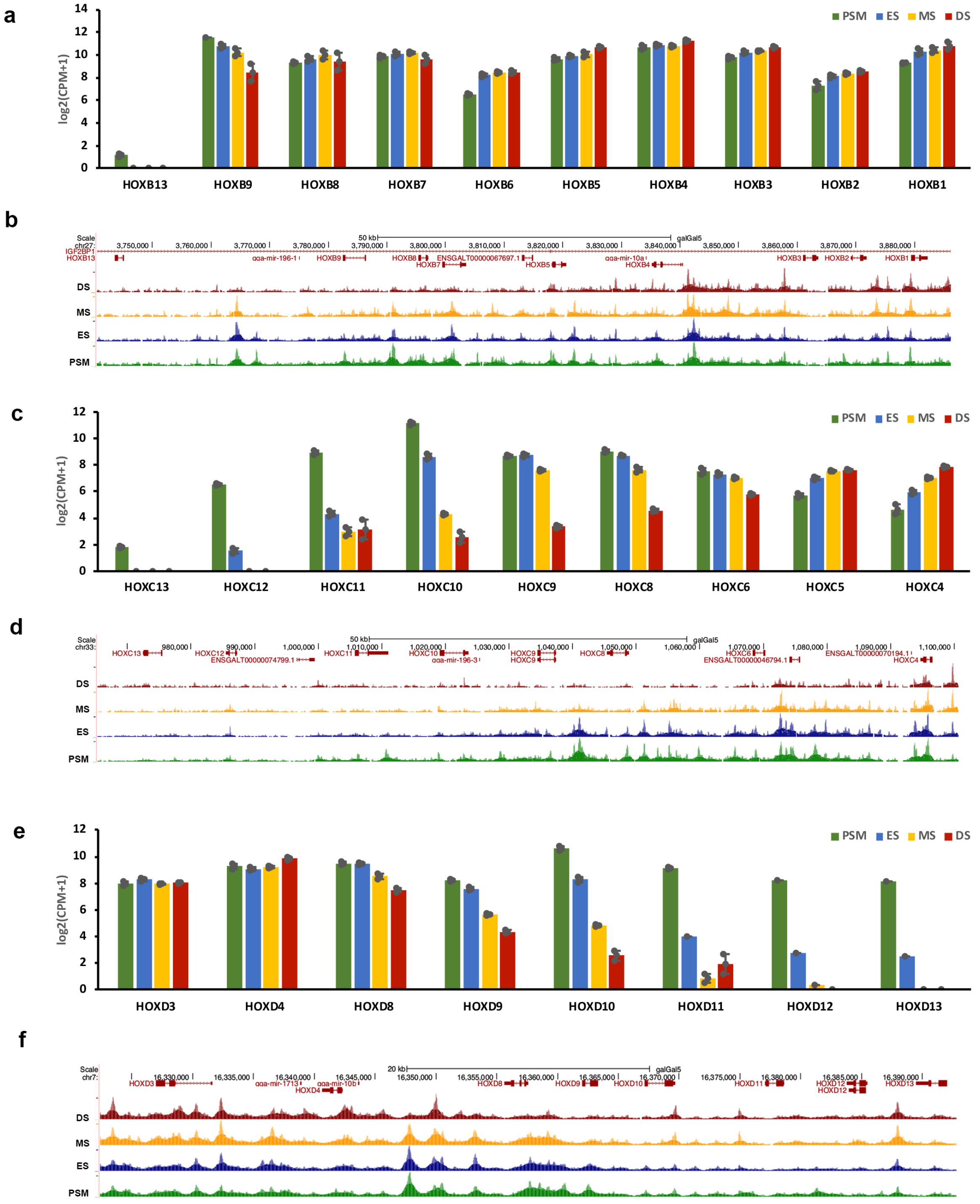

Supplementary Fig. 4: Chromatin accessibility and gene expression for *HoxB*, *HoxC* and *HoxD* clusters. a Gene expression from mRNA-seq for *HoxB* cluster (error bars = SEM, n = 3). **b** Genome browser views of ATAC-seq profile at *HoxB* cluster. **c** Gene expression from mRNA-seq for *HoxC* cluster (error bars = SEM, n = 3). **d** Genome browser views of ATAC-seq profile at *HoxC* cluster. **e** Gene expression from mRNA-seq for *HoxD* cluster (error bars = SEM, n = 3). **f** Genome browser views of ATAC-seq profile at *HoxD* cluster. PSM ATAC is shown in green, ES shown in blue, MS shown in yellow and DS in red.

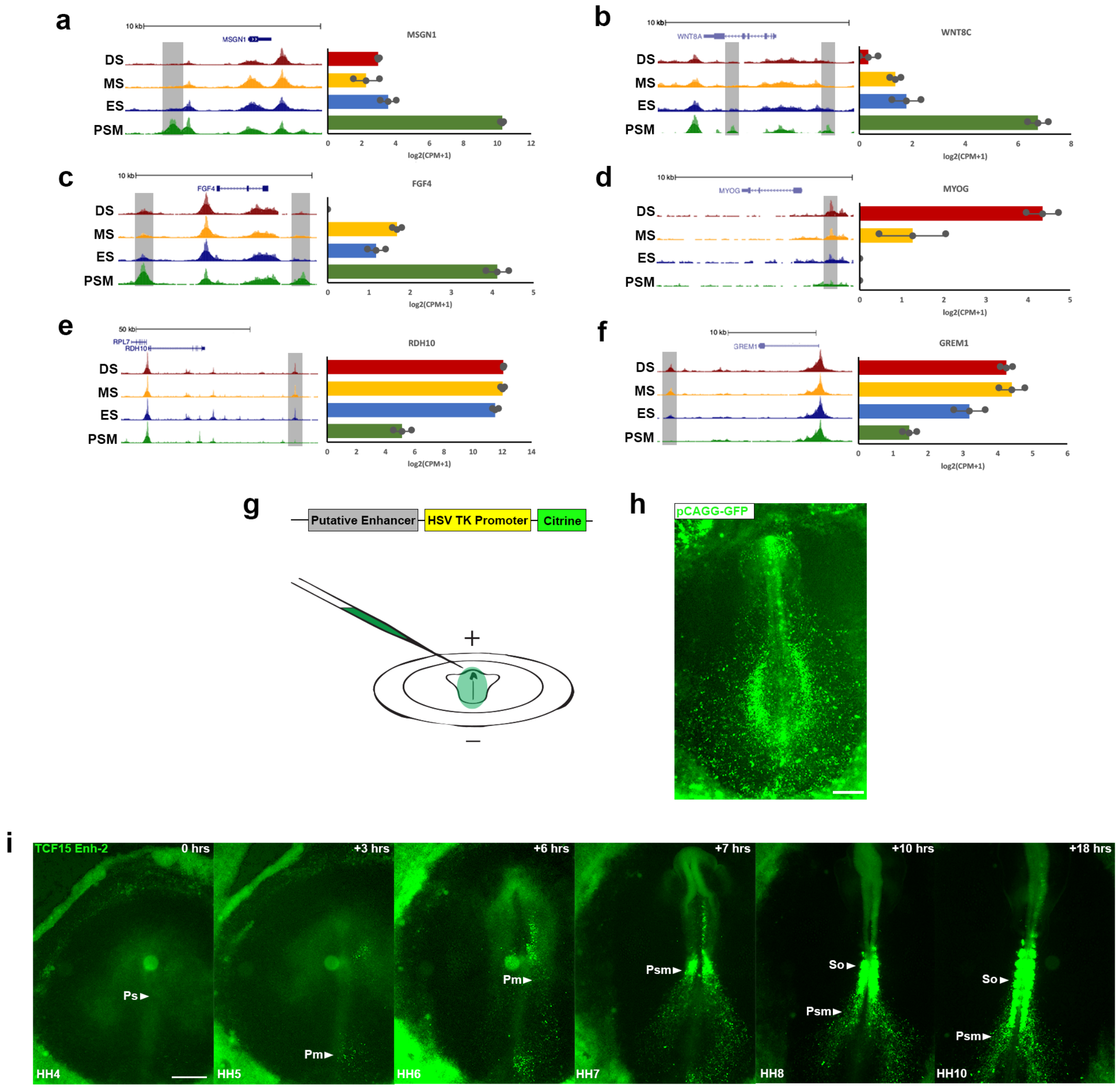

Supplementary Fig. 5: **Differential chromatin accessibility highlights putative CREs and time-lapse imaging reveals *TCF15* Enh-2 activity in prospective paraxial mesoderm and somites. a-f** Examples of ATAC-seq accessible regions that define PSM (*Msgn1*, *Wnt8c*, *Fgf4*) or DS (*MyoG*) or developing somites (*Rdh10*, *Grem1*). Gene expression from mRNA-seq (error bars = SEM, n = 3) is shown in the bar charts on the right. Grey bars are examples of differential accessible regions across the four samples flanking the gene locus. **g** Schematic representation of putative enhancer Citrine reporter construct. Injection of constructs into HH3+ chick embryos with positive electrode at the top and negative electrode at the bottom. **h** Electroporation of pCAGG-GFP at HH3+, embryo developed for 20 hours showing electroporation efficiency. **i** Still images from a time-lapse movie of *TCF15* Enh-2 with the primitive streak (Ps) indicated. Fluorescent activity first observed in paraxial mesoderm (Pm) at HH6 and continuous expression in the paraxial mesoderm (Psm) at HH7 prior to expression in somites (So) at HH8 and HH10. Scale bars = 500 microns

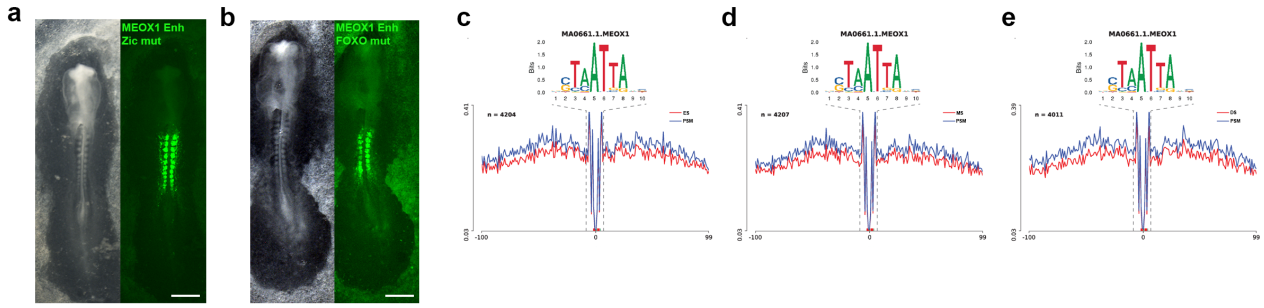

Supplementary Fig. 6: *MEOX1* enhancer reporter single mutants and MEOX1 footprints across different samples. a-b *MEOX1* Enh reporter constructs with mutation in either (**a**) ZIC (n = 4/4/) or (**b**) FOXO (n = 5/5) transcription factor binding sites show normal activity after electroporation into gastrula stage chick embryos. **c** Differential footprints for MEOX1 motif comparing PSM (blue) and ES (red), (**d**) PSM (blue) and MS (red) and (**e**) PSM (blue) and DS (red). Total number of footprints shown on each panel, n.

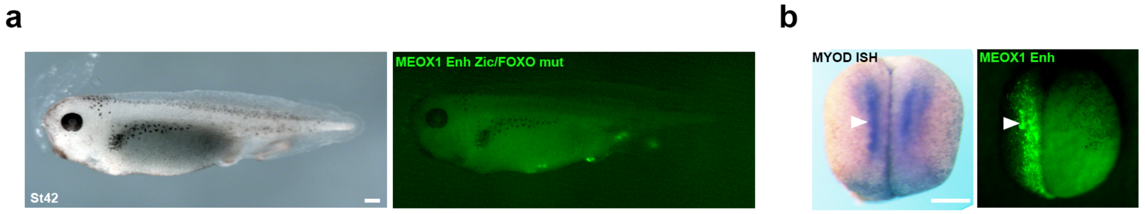

Supplementary Fig. 7: Testing avian putative Meox1 Enhancer in Xenopus. a *MEOX1* Enh with both Zic and FOXO mutations injected in Xenopus embryos. Activity is not observed in stage 42 embryos (n = 20/20). **b** *MEOX1* Enh shows expression similar to early paraxial mesoderm marker (*MYOD*) in gastrula-stage embryos. *MYOD* in situ hybridisation performed on same stage. Purple shows expression in paraxial mesoderm (white arrow heads, n = 10/10). Scale bars = 250 microns

Supplementary Table 1. Primers and sgRNAs.

Name of primer	Primer sequence
MEOX1 Enh F	TTTTTTCGTCTCgccaggCTTGCAGTCCTTTGATCCACC
MEOX1 Enh R	TTTTTTCGTCTCcaacagAGAGCCCAGA GTGGTTTTGC C
TCF15 Enh-1 F	TTTTTTCGTCTCgccaggACACACAGCAGCGGGTCGGTGCC
TCF15 Enh-1 R	TTTTTTCGTCTCcaacagGAGCAGGCAG GTGCCTGCAG CC
TCF15 Enh-2 F	TTTTTTCGTCTCgccaggTTGCACCTCGTGCTCAGGGTCTG
TCF15 Enh-2 R	TTTTTTCGTCTCcaacagTGGAGGAAGG TGTGTGCCAG CAG
Human MEOX1 Enh F	TTTTTTCGTCTCgccaggAATGTGGCTCCCTTCCTAGC
Human MEOX1 Enh R	TTTTTTCGTCTCcaacagCAGCCGGTAACAGGCAAGGT
MEOX1 gRNA-1 F	TTTCGTCTCcAGTCgCCAAGCCCATTTGTTGGACGgtttaGAGACGAAA
MEOX1 gRNA-1 R	TTTCGTCTCtaaacCGTCCAACAAATGGGCTTGGcGACTgGAGACGAAA
MEOX1 gRNA-2 F	TTTCGTCTCcAGTCgCAGAGAGTGCTGGGGCAATGgtttaGAGACGAAA
MEOX1 gRNA-2 R	TTTCGTCTCtaaacCATTGCCCCAGCACTCTCTGcGACTgGAGACGAAA
MEOX1 gRNA-3 F	TTTCGTCTCcAGTCgAGGGCAAACAAGCCCCCGCTgtttaGAGACGAAA
MEOX1 gRNA-3 R	TTTCGTCTCtaaacAGCGGGGGCTTGTTTGCCCTcGACTgGAGACGAAA
MEOX1 gRNA-4 F	TTTCGTCTCcAGTCgTCCTGACACTTCATTCCTGAgtttaGAGACGAAA
MEOX1 gRNA-4 R	TTTCGTCTCtaaacTCAGGAATGAAGTGTCAGGAcGACTgGAGACGAAA
TCF15 gRNA-1 F	TTTCGTCTCcAGTCgGGGATTTACGGGCTCTGCACgtttaGAGACGAAA
TCF15 gRNA-1 R	TTTCGTCTCtaaacGTGCAGAGCCCGTAAATCCCcGACTgGAGACGAAA
TCF15 gRNA-2 F	TTTCGTCTCcAGTCgGGCAAATGGTGAAGGCAAACgtttaGAGACGAAA
TCF15 gRNA-2 R	TTTCGTCTCtaaacGTTTGCCTTCACCATTTGCCcGACTgGAGACGAAA
TCF15 gRNA-3 F	TTTCGTCTCcAGTCgGGGGGGCCTTTTGCTCCTGAgtttaGAGACGAAA
TCF15 gRNA-3 R	TTTCGTCTCtaaacTCAGGAGCAAAAGGCCCCCCcGACTgGAGACGAAA
TCF15 gRNA-4 F	TTTCGTCTCcAGTCgGGGCTGTCCTCATGTGTAACgtttaGAGACGAAA
TCF15 gRNA-4 R	TTTCGTCTCtaaacGTTACACATGAGGACAGCCCcGACTgGAGACGAAA
Scrambled control gRNA F	TTTCGTCTCcAGTCgTGCAGTGCTTCAGCCGCTgtttaGAGACGAAA
Scrambled control gRNA R	TTTCGTCTCtaaacAGCGGCTGAAGCACTGCAcGACTgGAGACGAAA
MEOX1 Enh FOXO1 ZIC3 Mut F	GGGCCACCAGAATTCCTTCCTTCAGGAATGAAGTGTCAGG
MEOX1 Enh FOXO1 ZIC3 Mut R	CTGCTGCTGTCACTCAGCAGCCCAG
TCF15 Enh-2 RARA Mut F	TGGTTCCTTCAAACAGGGTCAGGGCCCCTCAGCAA
TCF15 Enh-2 RARA Mut R	ACCCTGTTTGAAGGAACCATTTGTCCTCCTGTGCAGAGCCCGTAAA
Human MEOX1 Enh FOXO1 ZIC3 Mut F	TCTGGCACCCCGGAG
Human MEOX1 Enh FOXO1 ZIC3 Mut R	GTGCCCGGCCTTCAT
cMEOX1 RT F	GAGATCGCTGTGAACCTGG
cMEOX1 RT R	CGCTTCCACTTCATCCTTCT
cMEOX2 RT F	AGCAGTAAACCTTGACCTCAC
cMEOX2 RT R	CATTCACCAGTTCCTTTTCCG
cPax3 RT F	AGCAGAGCAACTGGAAGAGC
cPax3 RT R	GGTGGTTGAAAGCCATCAGT
cGAPDH RT F	TCTCTGGCAAAGTCCAAGTG
cGAPDH RT R	TCACAAGTTTCCCGTTCTCAG
cFAT4 RT F	TGGCAGCTCAAGGAATCTTAG
cFAT4 RT R	GGCAGTGTCGGCAGTAATATC
cTGFB2 RT F	TTGATGTCACTGAGGCTGTAC
cTGFB2 RT R	CAAATCTTGCTTCAGGCTCC
cUNCX RT F	CCTGGCTGTAAGAGGAGACG
cUNCX RT R	CGGCTCGTTGAAAGAGAAAC
cTBX18 RT F	GTAATGCTGACTCCCCGGTA
cTBX18 RT R	GTGGACACGAGGCTGGTATT
cNKX3-2 RT F	GACGCAGGTGAAGATCTGGT
cNKX3-2 RT R	GGCAGGCAGTAGTAGGGGTA
MEOX1 Enh FOXO1 Mut F	AGGGCTGTTGTTTTT
MEOX1 Enh FOXO1 Mut R	AAAAACAACAGCCCTCCCCTCTTGCCCAA
MEOX1 Enh ZIC3 Mut F	GGCAAACAAGCCCCC
MEOX1 Enh ZIC3 Mut R	GGGGGCTTGTTTGCCCTCTGCATCT
