# Supplementary material for: Characterising open chromatin in chick embryos identifies cis-regulatory elements important for paraxial mesoderm formation and axis extension
Source: Nat Commun. 2021 Feb 19;12:1157. doi: 10.1038/s41467-021-21426-7 (PMC7895974; doi:10.1038/s41467-021-21426-7)
Supplement: Supplementary file 5 — Reporting Summary [file 41467_2021_21426_MOESM5_ESM.pdf]

nature research

Corresponding author(s): Andrea Münsterberg

Last updated by author(s): Jan 15, 2020

Reporting Summary

Nature Research wishes to improve the reproducibility of the work that we publish. This form provides structure for consistency and transparency in reporting. For further information on Nature Research policies, see our Editorial Policies and the Editorial Policy Checklist.

Statistics

For all statistical analyses, confirm that the following items are present in the figure legend, table legend, main text, or Methods section.

n/a	Confirmed	
☐	☒	The exact sample size (n) for each experimental group/condition, given as a discrete number and unit of measurement
☐	☒	A statement on whether measurements were taken from distinct samples or whether the same sample was measured repeatedly
☐	☒	The statistical test(s) used AND whether they are one- or two-sided<br>*Only common tests should be described solely by name; describe more complex techniques in the Methods section.*
☒	☐	A description of all covariates tested
☒	☐	A description of any assumptions or corrections, such as tests of normality and adjustment for multiple comparisons
☒	☐	A full description of the statistical parameters including central tendency (e.g. means) or other basic estimates (e.g. regression coefficient) AND variation (e.g. standard deviation) or associated estimates of uncertainty (e.g. confidence intervals)
☒	☐	For null hypothesis testing, the test statistic (e.g. F, t, r) with confidence intervals, effect sizes, degrees of freedom and P value noted<br>*Give P values as exact values whenever suitable.*
☒	☐	For Bayesian analysis, information on the choice of priors and Markov chain Monte Carlo settings
☒	☐	For hierarchical and complex designs, identification of the appropriate level for tests and full reporting of outcomes
☒	☐	Estimates of effect sizes (e.g. Cohen's d, Pearson's r), indicating how they were calculated

Our web collection on statistics for biologists contains articles on many of the points above.

Software and code

Policy information about availability of computer code

Data collection

- ATAC-seq and RNA-seq raw sequencing data files (fastq) were downloaded from Novogene and Earlham Institute servers.
- Whole mount embryo microscopy images were taken using Zeiss SV11 Microscope using Jenoptik ProgRes C5 camera and visualised using the ProgRes Capture Pro 2.10.0.1 imaging software.
- Fertilised wild-type chicken eggs were obtained from Henry Stewart & Co (Norfolk)

Data analysis

ATAC-seq Processing

Adaptors were removed from raw paired-end sequencing reads and trimmed for quality using Trim Galore! (v.0.5.0) a wrapper tool around Cutadapt and FastQC. Default parameters were used. Quality control (QC) was performed before and after read trimming using FastQC (v.0.11.6) and no issues were highlighted from the QC process. Subsequent read alignment and post-alignment filtering was performed in concordance with the ENCODE project's "ATAC-seq Data Standards and Prototype Processing Pipeline" for replicated data (https://www.encodeproject.org/atac-seq/). In brief, reads were mapped to the chicken genome galGal5 assembly using bowtie2 (v.2.3.4.2). The resultant Sequence Alignment Map (SAM) files were compressed to the Binary Alignment Map (BAM) version on which SAMtools (v.1.9) was used to filter reads that were unmapped, mate unmapped, not primary alignment or failing platform quality checks. Reads mapped as proper pairs were retained. Multi-mapping reads were removed using the Python script assign_multimappers provided by ENCODE's processing pipeline and duplicate reads within the BAM files were tagged using Picard MarkDuplicates (v.2.18.12) [http://broadinstitute.github.io/picard/] and then filtered using SAMtools. For each step, parameters detailed in the ENCODE pipeline were used. From the processed BAM files, coverage tracks in bigWig format were generated using deepTools bamCoverage (v 3.1.2) and peaks were called using MACS2 (v.2.1.1) (parameters -f BAMPE -g mm -B --nomodel --shift -100 --extsize 200) . Coverage tracks and peaks (narrow peak format) were uploaded to the UCSC Genome Browser as custom tracks for ATAC-seq data visualization.

Differential Accessibility and Footprinting

Analysis of ATAC-seq for differential accessibility was carried out in R (v.3.5.1) using the DiffBind package (v.2.8.0) with default parameter settings. Differential accessibility across samples was calculated using the negative binomial distribution model implemented in DEseq2

(v1.4.5). Computational footprinting analysis was conducted across samples using HINT-ATAC which is part of the Regulatory Genomic Toolbox (v.0.12.3) also using default parameter settings and the galGal5 genome.

RNA-seq Differential Expression Analysis
Adaptors were removed from raw paired-end sequencing reads and trimmed for quality using Trim Galore! (v.0.5.0) using default parameters. Quality control was performed before and after read trimming using FastQC (v.0.11.6) and no data quality issues were identified after checking the resultant QC reports. Processed reads were mapped to galGal5 cDNA using kallisto (v.0.44.0). Resultant quantification files were collated to generate an expression matrix. Differential expression, GO term and pathway analyses were then conducted using DESeq2 and default settings within the iDEP (v.9.0) web interface.GO term analysis used PGSEA method for GO Biological Process with a minimum of 15 and maximum of 2000 geneset and <0.2 FDR.

Image analysis
Sections were visualized on an Axioscope with Axiovision software (Zeiss). Whole mount embryos were photographed on a Zeiss SV11 dissecting microscope with a Micropublisher 3.5 camera and acquisition software or Leica MZ16F using Leica Firecam software. Live imaging datasets were analysed in FIJI/ImageJ.

For manuscripts utilizing custom algorithms or software that are central to the research but not yet described in published literature, software must be made available to editors and reviewers. We strongly encourage code deposition in a community repository (e.g. GitHub). See the Nature Research guidelines for submitting code & software for further information.

Data

Policy information about availability of data

All manuscripts must include a data availability statement. This statement should provide the following information, where applicable:
- Accession codes, unique identifiers, or web links for publicly available datasets
- A list of figures that have associated raw data
- A description of any restrictions on data availability

ATAC-seq and RNA-seq raw sequencing data for this study is stored at the Sequence Read Archive (SRA) using the BioProject accession: PRJNA602335. All other data supporting the findings of this study are available from the corresponding author on reasonable request.

Field-specific reporting

Please select the one below that is the best fit for your research. If you are not sure, read the appropriate sections before making your selection.

☒ Life sciences ☐ Behavioural & social sciences ☐ Ecological, evolutionary & environmental sciences

For a reference copy of the document with all sections, see nature.com/documents/nr-reporting-summary-flat.pdf

Life sciences study design

All studies must disclose on these points even when the disclosure is negative.

Sample size	ATAC-seq or RNA-seq experiment was done on a pool of 4 somites per tissue sample per replicate. 3 embryos were used for ATAC-seq and RNA-seq sample collection. For CRISPR experiments individual embryo samples were collected after electroporation to collect for RNA and subsequently qPCR. A minimum of 4-5 embryos were electroporated for each condition.
Data exclusions	n/a
Replication	ATAC-seq and RNA-seq were carried out in experimental triplicates. Pearson score for ATAC-seq and RNA-seq experiments showed high correlation across replicates that clusters together (See PCA plots).
Randomization	n/a
Blinding	n/a

Reporting for specific materials, systems and methods

We require information from authors about some types of materials, experimental systems and methods used in many studies. Here, indicate whether each material, system or method listed is relevant to your study. If you are not sure if a list item applies to your research, read the appropriate section before selecting a response.

Materials & experimental systems

n/a	Involved in the study	
	x	Antibodies
x		Eukaryotic cell lines
x		Palaeontology and archaeology
	x	Animals and other organisms
x		Human research participants
x		Clinical data
x		Dual use research of concern

Methods

n/a	Involved in the study	
x		ChIP-seq
x		Flow cytometry
x		MRI-based neuroimaging

Antibodies

Antibodies used	Primary antibody for rabbit anti-GFP (1:200, Torrey Pines Biolabs Cat no. TP401) and secondary antibody AlexaFluor-568-conjugated donkey anti-rabbit IgG (1:500, ThermoFisher Cat no. A21206).
Validation	For anti-GFP, the antibody has been successfully used in immunohistochemistry in published papers specifically in chick embryos (Ling ITC and Sauka-Spengler T, Nat Cell Biol 2019 Dec;21(12):1504-1517. doi: 10.1038/s41556-019-0428-9.)

Animals and other organisms

Policy information about studies involving animals; ARRIVE guidelines recommended for reporting animal research

Laboratory animals	chick embryos are not governed by the Animals Scientific Procedures Act up to embryonic day 14, we used embryos up to E4
Wild animals	n/a
Field-collected samples	n/a
Ethics oversight	All experiments were performed on chicken embryos younger than 12 days of development, and as such were not regulated by the Animals (Scientific Procedures) Act 1986

Note that full information on the approval of the study protocol must also be provided in the manuscript.
