# Supplementary material for: Characterising open chromatin in chick embryos identifies cis-regulatory elements important for paraxial mesoderm formation and axis extension
Source: Nat Commun. 2021 Feb 19;12:1157. doi: 10.1038/s41467-021-21426-7 (PMC7895974; doi:10.1038/s41467-021-21426-7)
Supplement: Supplementary file 6 — Description of Additional Supplementary Files [file 41467_2021_21426_MOESM6_ESM.pdf]

Title: Supplementary Movie 1

Description: Time-lapse of TCF15 Enh2 Citrine-reporter construct electroporated into chick gastrula stage embryos. Brightfield and darkfield views of *TCF15* enhancer activity. In each panel anterior is to the left, posterior is to the right.

Title: Supplementary Movie 2

Description: Time-lapse of MEOX1 Enh Citrine-reporter construct electroporated into chick gastrula stage embryos. Brightfield and darkfield views of *MEOX1* enhancer activity. In each panel anterior is to the left, posterior is to the right.
